# Fabrication of P/N/B-Based Intumescent Flame-Retardant Coating for Polyester/Cotton Blend Fabric

**DOI:** 10.3390/ma15186420

**Published:** 2022-09-15

**Authors:** Wei-Lin He, Yi-Ting Huang, Liang Gu, Ji-Cheng Shen, Xian-Wei Cheng, Jin-Ping Guan

**Affiliations:** 1 Key Laboratory of Flame Retardancy Finishing of Textile Materials (CNTAC), College of Textile and Clothing Engineering, Soochow University, Suzhou 215123, China; 2 Suzhou Haitai Textile Co., Ltd., Suzhou Knitting Industrial Park, Suzhou 215228, China

**Keywords:** polyester/cotton blend fabric, flame retardant, intumescent, surface coating, phytic acid

## Abstract

Polyester/cotton (T/C) blend fabrics are highly flammable due to the particular “scaffolding effect”. In this work, an intumescent flame retardant (IFR) agent containing P, N, and B was designed and synthesized using bio-based phytic acid, pentaerythritol, boric acid, and urea. The IFR compounds were deposited onto a T/C blend fabric by the surface-coating route. The chemical structure of IFR agent and its potential cross-linking reactions with T/C fibers were characterized. The morphology, thermal stability, heat-release ability, flame retardancy, and mechanism of coated T/C blend fabrics were explored. The self-extinguishing action was observed for the coated T/C blend fabric with a weight gain of 13.7%; the limiting oxygen index (LOI) value increased to 27.1% versus 16.9% for a pristine one. Furthermore, the intumescent flame retardant (IFR) coating imparted T/C blend fabrics with high thermal stability and significantly suppressed heat release by nearly 50%. The char residue analyses on morphology and element content confirmed the intumescent FR action for coated T/C blend fabrics. The prepared IFR coating has great potential to serve as an eco-friendly approach for improving the flame retardancy of T/C blend textiles.

## 1. Introduction

Polyester/cotton (T/C) blend fabrics have found their wide application in soldier and firefighter uniforms, bedding, and upholstery fabrics. This can be attributed to their comfortability, breathability, quick-drying performance, high elastic recovery, and wrinkle resistance performance, all advantages of polyester and cotton [1,2,3]. However, raw cotton and polyester fabrics are highly flammable materials that could bring serious fire hazards, while the combustion process of T/C blend fabrics is severe due to the unique “wick effect” [4,5]. During burning, cotton degrades first to provide fuel, and polyester degrades in the following stage to continuously provide fuel for burning. Furthermore, the melted polyester would conglutinate on the charred cotton skeletons and could not generate molten droplets from the fire zone, providing more energy and fuel. The burning of a T/C blend fabric would generate a large mass of poisonous gas and toxic smoke. These cause greater fire hazards and greatly limit their application. Therefore, it is necessary and challenging to impart an efficient flame retardant (FR) ability to a T/C blend fabric.

In this context, many FR solutions involving the application of halogen-, phosphorus-, and mineral-containing additives and back-coatings have been exploited for improving the flame retardancy of cotton and T/C blend fabrics [6,7]. Furthermore, the eco-friendly and high effective FR approaches are under consideration, after the recognition of the toxic features of certain halogen-based FR agents and some commercial phosphorus-based FR systems with potential formaldehyde release during service [8,9]. The application of a formaldehyde-free crosslinking agent for Pyrovatex CP New and for other FR agents, such as poly(amidoamine) dendrimer [10,11], and the plasma grafting of FR chemicals, such as Hexamethyldisiloxane [12], showed high potential to address this issue. The recently developed formaldehyde-free FR technique of grafting reactive P-N containing a FR system onto cellulose fibers also displayed high potential to improve the flame retardancy and washing resistance of cotton fabric [13]. It is noticed that an FR approach that can catalyze the formation of a physical barrier on a material’s surface can be considered as an effective strategy to replace the toxic halogen-based FR approaches. An intumescent FR (IFR) system, as a new type of FR approach, has attracted great interest due to its environmental friendliness and high efficiency. An IFR system could generate a uniform intumescent char layer on a material’s surface, which functions mainly in the condensed phase. At the same time, the noncombustible nitrogen-based species can function in the gas phase [14,15].

Furthermore, the recently developed natural compounds derived from animals and plants such as proteins [16], deoxyribonucleic acid [17], aromatic tannins [18], chitosan, and lignin [19] also attracted great interest [20,21]. Phytic acid (PA) represents an organic phosphoric acid with an eco-friendly feature and high phosphorus content (28 wt%). PA is originated from the seeds, roots, and stems of plants, and it has been widely applied in antioxidants, anticancer agents, biosensors, and cation exchange resins due to its environmental benignity and special inositol hexaphosphate structure [22,23]. Bio-based PA has been applied as the anionic counterpart to fabricate the IFR coating on cotton, polyester, and wool textile surfaces, through layer-by-layer assembly together with cationic counterparts such as chitosan, polyethyleneimine, and silica sols [24,25]. Besides, to develop durable FR cellulose textiles, such as cotton and lyocell fabrics, the preparation of ammonium phytate and covalent grating with cellulose fibers aroused great interest [26,27]. However, there are limited explorations into PA-based FR systems for reducing the flammability of T/C blend fabrics.

Boron compounds such as boric acid and borax are well-known FRs for cotton fabric. They also have the features of low toxicity and a lack of odor or color, and, thus, they can be used to prepare halogen-free FR chemicals for polymers [28]. The boron-containing coatings formed during combustion could serve as thermal and oxygen barriers by intumescent action, and they are able to promote char formation and prevent further fire propagation via their acidic properties [29].

In this study, a novel P/N/B-containing IFR agent was designed and prepared by using PA, boric acid, pentaerythritol, and urea through ester reaction. PA and boric acid could serve as the acid source, the pentaerythritol portion could function as the carbon source, and the ammonium groups acted as the gas source. The IFR agent was applied to improve the flame retardancy of a T/C blend fabric by the surface-coating approach. Furthermore, the possible chemical structure of IFR agent was characterized using nuclear magnetic resonance (NMR). For the coated T/C blend fabrics, the thermal stability and heat-release property were evaluated; the FR ability and potential FR mechanism were also explored.

## 2. Experimental Section

### 2.1. Materials

A polyester/cotton (T/C) blend fabric (blend ratio 80:20, grammage 94 g/m^2^) was purchased from Hebei Longma Textile Co., Ltd., Shijiazhuang, Hebei, China. Phytic acid (70 wt%), boric acid, urea, pentaerythritol, and sodium hypophosphite were purchased from Shanghai Macklin Biochemical Co., Ltd., Shanghai, China. Dicyandiamide, ethanol, toluene, and deuterium oxide were purchased from Jiangsu Chinasun Specialty Products Co., Ltd., Changshu, Jiangsu, China.

### 2.2. Preparation and Characterization of IFR

**Figure 1:**
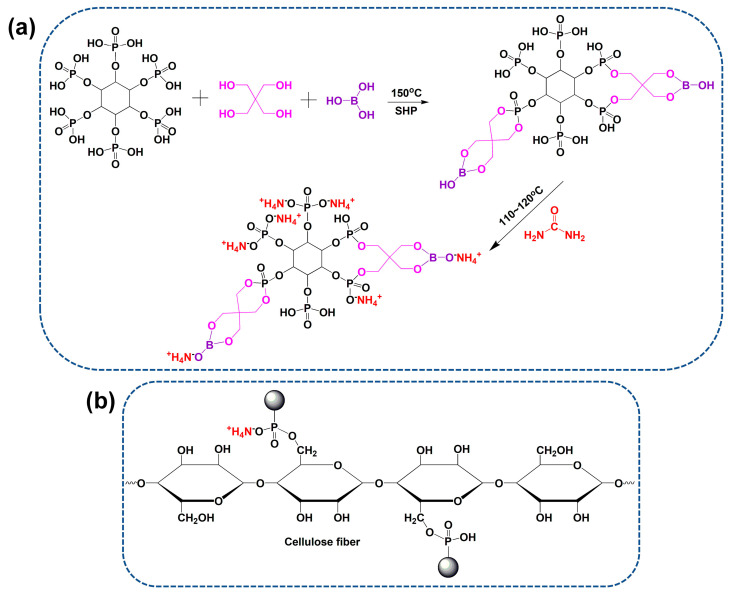
Synthesis route of IFR agent (**a**) and its cross-linking action with cellulose fiber (**b**).

Firstly, PA (0.02 mol, 18.9 g), pentaerythritol, and boric acid were put into a three-necked flask with a molecular ratio of 1:2:2. Sodium hypophosphite (0.02 mol, 1.8 g) was added as catalyst, and 20 mL toluene was added as solvent. The reaction was performed at 150 °C for 2.5 h under magnetic stirring. Next, urea (0.12 mol, 7.8 g) was added into the flask after being cooled down at 110 °C, the reaction was continued for 1.5 h, and then reacted at 120 °C for 0.5 h, yielding a viscose and transparent liquid. The toluene was removed through vacuum distillation, and the crude IFR product was washed using ethanol. The synthetic reaction of IFR is shown in Figure 1a. The possible chemical structure of IFR agent was also confirmed using ^1^H and ^31^P NMR spectra [23,25]. ^1^H NMR (D_2_O, 400 MHz) δ (ppm): 7.61 (8H, B-O-CH_2_), 6.32 (8H, P-O-CH_2_), 4.80 (deuterium oxide), 3.98–3.20 (6H, CH, inositol hexane ring). ^31^P NMR (D_2_O, 400 MHz) δ (ppm): 7.02, 1.46, 0.14, −10.22.

### 2.3. Preparation of Coated T/C Blend Fabrics

A series concentration of IFR solutions (100~400 g/L) were prepared using the synthesized product. Dicyandiamide was added to the solutions as a catalyst for ammonium phosphate groups and hydroxyl groups of cellulose fiber (Figure 1b) at a concentration of 50 g/L. First, the T/C blend fabrics were dipped into the IFR solutions at the liquor ratio of 1:25, heated to 60 °C, and kept for 30 min under oscillating conditions. Then, the T/C blend fabrics were padded using a lab padder, giving a wet pick-up of 100 ± 5%. The T/C blend fabrics were pre-dried and cured at 160 °C for 3 min. Finally, the T/C blend fabrics were rinsed and air-dried. The weight gain was determined by weighing samples before and after the coating treatment. The T/C blend fabrics treated by 200 and 400 g/L IFR were denoted as T/C-1 and T/C-2, respectively, in the following section.

### 2.4. Characterizations

The nuclear magnetic resonance (NMR) analysis was conducted on the Bruker Avance III 400 MHz spectrometer (Bruker BioSpin GmbH, Rheinstetten, Germany) using deuterium oxide as solvent. The Fourier-transform infrared spectroscopy (FTIR) analysis was conducted on Nicolet iS50 FTIR Spectrometer (Thermo Fisher Scientific Inc., Waltham, MA, USA). The T/C blend fabrics and their residues were firstly sputter-coated with a gold layer, and then their surface morphologies were observed on Hitachi S4800 field-emission and Hitachi TM3030 scanning electron microscope (SEM), with matching energy dispersive spectroscopy (EDS) (Hitachi High Technologies America, Inc., Schaumburg, IL, USA). Thermogravimetry (TG) analysis was investigated on the TA Q600 SDT thermal analyzer (TA Instruments, New Castle, DE, USA). The heat release property was, respectively, evaluated by FTT0001 pyrolysis combustion flow calorimetry (PCFC) (Fire Testing Technology Ltd., East Grinstead, UK) according to ASTM D7309; each sample was tested three times, and the average combustion data were used as the results. Limiting oxygen index (LOI) test was conducted according to GB/T 5454-1997, using FTT0080 oxygen index tester (Fire Testing Technology Ltd., East Grinstead, UK) with a propane burner. The vertical flame test was conducted using YG815B vertical flammability tester (Ningbo Textile Instrument Factory, Ningbo, Zhejiang, China) according to GB/T 5455-2014, by applying a 40 mm propane flame for 12 s; the corresponding burning classification was evaluated according to GB 8624-2012. The washing durability test of the IFR-coated T/C blend fabric was carried out according to AATCC-61-2013. The tensile strength was measured using Instron 3365 (Illinois Tool Works Inc., HighWycombe, Buckinghamshire, UK) according to ISO 13934-1-2013, and the test was performed five times for each sample. The air permeability of the fabric was tested by using the YG461G fully automatic permeability instrument (Wenzhou Darong Textile Instrument Co., Ltd., Wenzhou, Zhengjiang, China) according to GB/T 5453-1997, and the test was performed five times for each sample. The bending length of the fabrics was evaluated according to GB/T 18318.1–2009 by YG(B)022D automatic textile stiffness machine (Wenzhou Darong Textile Instrument Co., Ltd., Wenzhou, Zhengjiang, China), and the test was performed ten times for each sample.

## 3. Results and Discussion

### 3.1. FTIR and Morphology Analyses

**Figure 2:**
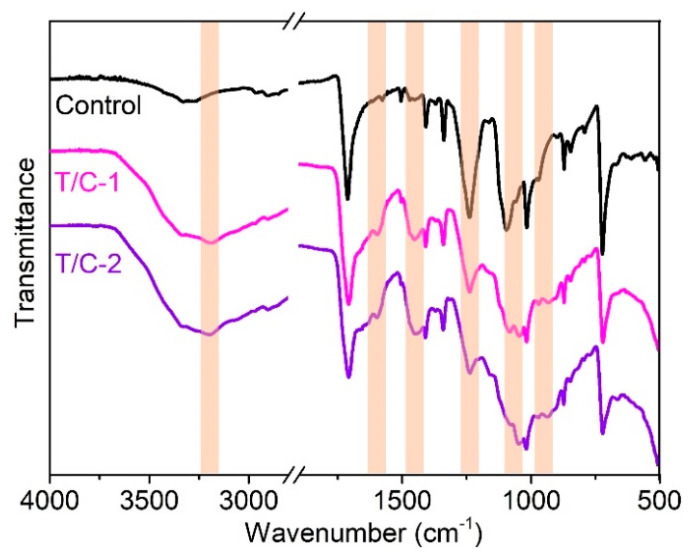
ATR-FTIR spectra of the coated T/C blend fabrics.

The chemical structures of the coated T/C blend fabrics are characterized using the FTIR. As shown in Figure 2, the coated T/C blend fabrics showed similar spectra versus the pristine one, except for several new absorptions. The new peaks, occurring at around 3200 and 1597 cm^−1^, should be ascribed to the stretching and bending vibrations of NH_4_^+^ structures, respectively [27]. The new peak at 1448 cm^−1^ belongs to the stretching vibration of B-O-C groups [30,31]. The peaks at 1237, 1045, and 963 cm^−1^ are assigned to the P=O and P-O groups [26,27]. Besides, the weak peak difference at around 1165 cm^−1^ corresponds to the P-O-C bonds formed during the coating process. These results agree with the cross-linking reactions between cellulose fibers and IFR agent displayed in Figure 1b.

**Figure 3:**
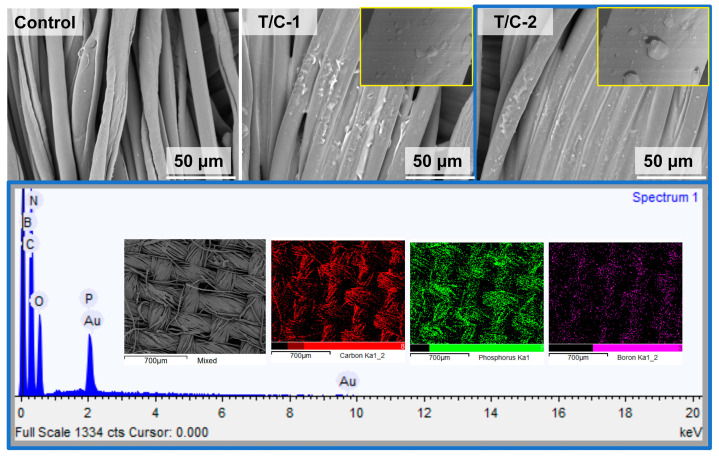
SEM images and EDS mapping of coated T/C blend fabrics.

Figure 3 shows the SEM images of the coated T/C blend fabrics. The pristine T/C blend fabrics displayed a smooth surface, while the regular cylindrical fibers and the fibers with a natural twist in appearance correspond to the polyester and cotton fibers, respectively. After the IFR coating, the fibers’ surface was homogeneously covered by the IFR coating. The integrity of the fiber structure was well-preserved, although it showed disappeared gaps between the close two fibers. Furthermore, according to the EDS mapping, the P and B elements were also found to be homogeneously dispersed onto the fibers, other than the C and O elements. These results above confirmed that the IFR coating was finely introduced on the T/C blend fabrics.

### 3.2. Thermal Stability

**Figure 4:**
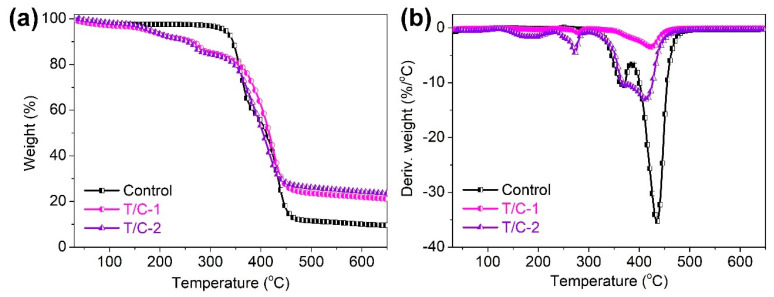
TG (**a**) and DTG (**b**) curves of coated T/C blend fabrics in nitrogen.

The thermal stability of the coated T/C blend fabrics was evaluated by TG analysis in nitrogen. Figure 4 shows the TG and derivative TG (DTG) curves of the coated T/C blend fabrics. Table 1 lists the corresponding degradation data including the *T_5%_*, *T_max1_*, and *T_max2_* values (temperatures at 5% weight loss and the first and second peak weight loss, respectively) and char residue at 650 °C. Two degradation peaks were found for the T/C blend fabrics in nitrogen. The first peak at around 369.8 °C corresponds to the degradation of cellulose fiber, and the second peak at around 434.7 °C is ascribed to the degradation of polyester fiber [3,32]. The coated T/C blend fabrics had a similar degradation trend versus the pristine one. However, the IFR-coated T/C blend fabrics displayed the anticipated degradation behavior versus the pristine one, as confirmed by the significantly lowered *T_5%_* value listed in Table 1. This result was also confirmed by the reported literature related to the FR modification of cellulose textiles using PA-based systems [26,27]. For the coated T/C blend fabrics, the small degradation peak that occurred at around 272.6 °C should be ascribed to the decomposition of the IFR agent. The formed phosphoric acid, polyphosphoric acid, and borax compounds in the early degradation stage were beneficial to promote the generation of the thermally stable char layer. The thermally protective char layer could retard heat and the oxygen attack on the substrates below and enhanced the thermal stability of the coated T/C blend fabrics. As a result, the coated T/C samples had higher char residue at the end of the degradation. As confirmed by TG analyses, the increased char residue is also responsible for the lowered number of flammable species upon degradation.

### 3.3. Heat Suppression Performance

**Figure 5:**
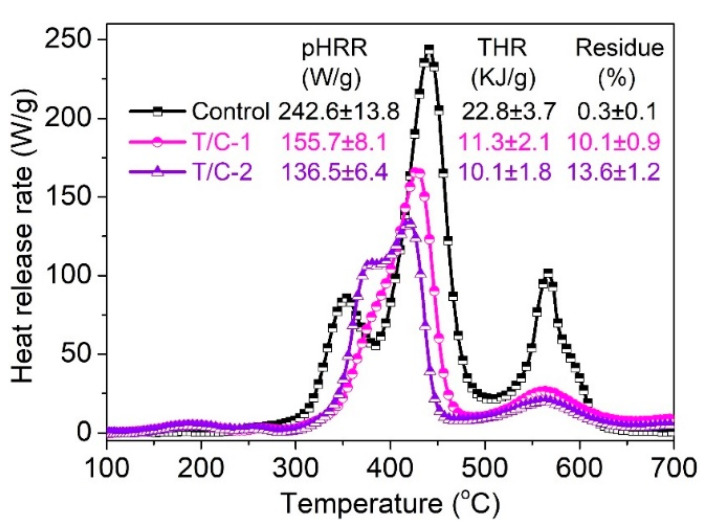
HRR curves of coated T/C blend fabrics.

Figure 5 displays the heat release rate (HRR) curves of coated T/C blend fabrics and lists the corresponding parameters such as peak HRR (pHRR), total heat release (THR), and char residue. The pristine T/C blend sample displayed multiple peaks due to the presence of different organic matters, corresponding to the results of TG analysis. The pHRR value of 242.6 W/g and THR value of 22.8 KJ/g were achieved for the pristine sample. It is obvious that the pHRR and THR values of coated T/C blend fabrics are much lower compared with those of the pristine one. Specifically, the THR of T/C-1 and T/C-2 was reduced by 50.4% and 55.7%, respectively. The pHRR value also showed a similar reduction trend with THR. The heat-suppression ability should be ascribed to the protective action of the thermally stable physical barrier, for inhibiting the mass and heat transfer between the condensed phase and gas phase. The less than complete combustion of the coated T/C blend fabrics, as demonstrated by the increased char amount, also contributed to the lowered heat release. As described above, the heat release of the T/C blend fabrics were significantly suppressed.

### 3.4. Flame Retardancy

**Figure 6:**
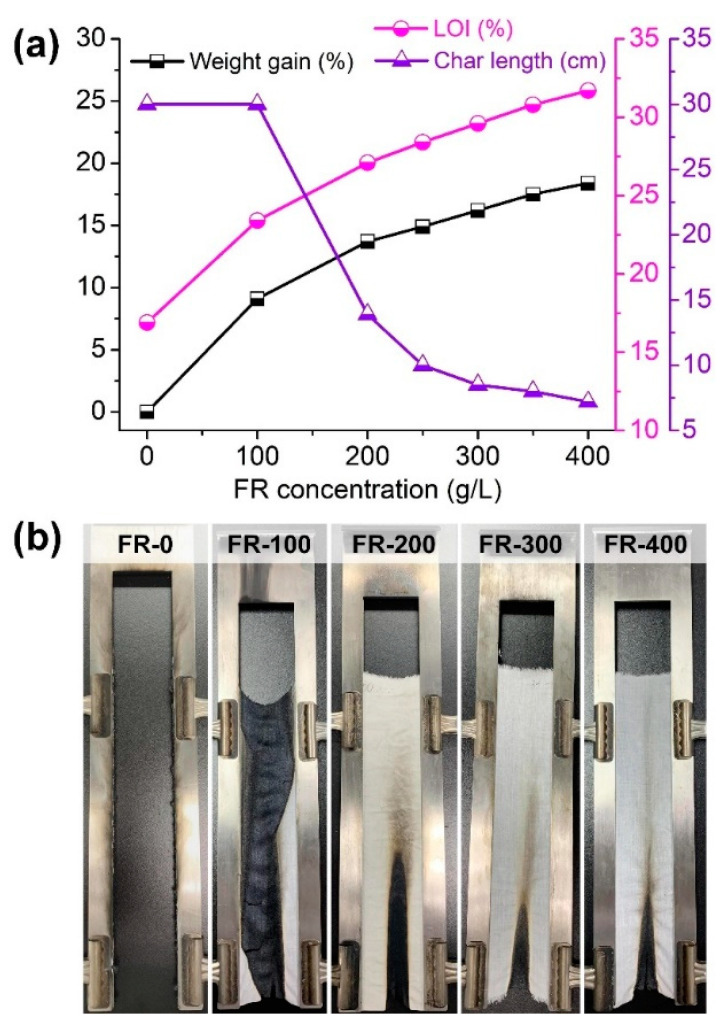
Weight gain and flame retardancy of the coated T/C blend fabrics (**a**) and the corresponding photos after vertical burning test (**b**).

The vertical burning and LOI tests were applied to explore the flame retardancy of the coated T/C blend fabrics responding to an open flame. Figure 6a shows the weight gain and flammability parameters of the coated T/C blend fabrics at various IFR concentrations, and Figure 6b displays the corresponding photos after a vertical burning test. The uncoated T/C blend fabric was ignited easily and then burned violently and completely, leaving several melted residues at device boundary with a char length of 30 cm. As a comparison, the coated T/C blend fabric with 100 g/L IFR preserved the char residue with the textile structure, although it burned completely and obtained a char length of 30 cm.

Higher IFR concentration imparted better flame retardancy to the T/C blend fabrics due to the higher weight gain. The fabrics coated with more IFR agent than 200 g/L self-extinguished after the removal of the ignition source, without an after-flame or after-glow effect. They had a significantly reduced char length of lower than 13.9 cm, reaching the B_1_ level. The burner was not able to ignite the char region again. Besides, the LOI of the coated T/C blend fabrics increased accordingly with weight gain, displaying the further enhanced FR ability. The LOI of T/C-1 and T/C-2 increased to 27.1% and 31.7%, respectively, versus 16.9% for the pristine one. However, the coated T/C blend fabric (T/C-2) burned completely after 10 washing cycles, which should be ascribed to the low cotton component and insufficient cross-linking of the IFR coating onto the T/C blend fabrics.

### 3.5. Char Residue Analyses

**Figure 7:**
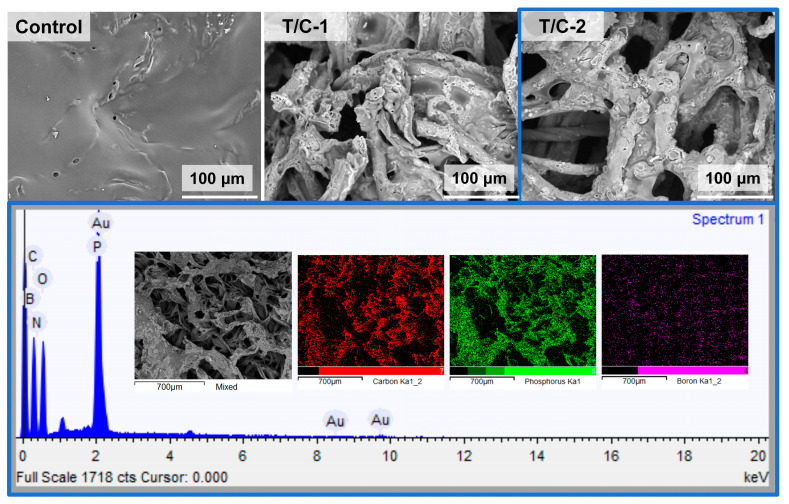
SEM images and EDS mapping of T/C char residues from vertical burning test.

The pristine T/C blend fabric burned drastically due to the “scaffolding effect” of polyester and cotton skeletons. As shown in Figure 7, the melted residue of the T/C blend sample displayed a smooth surface with some holes, probably because of the burning competition of cellulose with polyester, while the coated T/C blend samples displayed the damaged fibers on their char residues. Furthermore, the melted component was observed to be adhered on the surface of fiber residue. It is supposed that the cotton component maintained the integrity of fiber structure, and the molten droplets produced by the polyester component clung to the cotton fiber residue, preventing the so-called “runaway effect”. The whole thermal stability of the T/C blend fabric was also improved by the IFR coating. This corresponds to the lower fire hazards of the coated T/C blend fabrics.

Additionally, the char residues of the coated T/C blend samples also showed the homogeneously distributed P and B elements, according to the EDS mapping (Figure 7). Interestingly, the burned T/C residues had higher P and B content than the unburned T/C blend fabrics. For example, the P and B elements of the T/C-2 sample enriched from 3.2% and 4.4% before burning to 10.3% and 10.5% after burning, respectively. These results above demonstrate that the P and B elements worked and then were reserved in a condensed phase after combustion. The IFR coating could improve the flame retardancy of the T/C blend fabric by the condensed phase mechanism.

### 3.6. Physical Performance

The air permeability, tensile strength, and handle of the coated T/C blend fabrics were evaluated. As shown in Table 2, the coated T/C blend fabrics had slightly decreased air permeability versus that of the control, because of the grafting of the IFR agent onto the fabric surface. According to the reports applying ammonium phytate as a durable FR approach for cotton fabric, the covalent crosslinking of ammonium phytate with cotton fiber significantly damaged the mechanical performance of the cotton fabric [26,27]. However, as shown in Table 2, the mechanical performance of the T/C blend fabric was not influenced by the IFR coating. It is supposed that less IFR agent could combine with the T/C blend fabrics due to the low cellulose content, corresponding to the insufficient washing resistance of the IFR coating on the T/C blend fabrics. Besides, the FR treatment also modestly impacted the handle of the T/C blend fabric, as indicated by the slightly increased bending length and flexural rigidity.

## 4. Conclusions

The present study prepared a novel P/N/B-containing IFR agent for improving the flame retardancy of the T/C blend fabric by the surface-coating technique. The chemical structure of IFR agent was confirmed by ^1^H and ^31^P NMR. The coated T/C blend fabrics possessed higher LOI values than 27.1% and showed a self-extinguishing ability at a weight gain of 13.7%. Higher weight gain contributed to the further improved flame retardancy of the coated T/C blend fabrics. TG analysis suggested the catalytic ability of the IFR agent to promote the char-forming T/C blend fabric. Besides, the char region of the burned T/C samples preserved the fiber structure and intumescent bubbling structures with high P and B content, and the char layer could impede the heat and fuel transfer to the condensed phase. The IFR coating exerted little influence on the physical properties of the coated T/C blend fabrics. This should be ascribed to the lower grafting extent of the IFR coating on T/C blend fabrics with low cellulose content. Correspondingly, the IFR coating had undesirable washing resistance on the T/C blend fabrics. This study offers a sustainable IFR coating for improving the flame retardancy of the T/C blend fabrics. Continuous explorations are ongoing to improve the durability of IFR coating, to broaden its potential application fields.

## Figures and Tables

**Table 1:** TG data of coated T/C blend fabrics in nitrogen.

Samples	*T_5%_* (°C)	*T_max1_* (°C)	*T_max2_* (°C)	Residue at 650 °C (%)
Control	329.4	369.8	434.7	9.6
T/C-1	175.4	—	414.4	21.1
T/C-2	181.4	—	423.9	23.5

**Table 2:** Air permeability, tensile strength, bending length, and flexural rigidity of the coated T/C blend fabrics.

Samples	Air Permeability (mm/s)	Tensile Strength (N)	Bending Length (mm)	Flexural Rigidity (mN cm)
Control	725.5 ± 11.3	613.5 ± 10.5	15.1 ± 1.2	3.3 ± 0.1
T/C-1	699.8 ± 10.9	611.1 ± 8.7	15.9 ± 1.1	3.8 ± 0.2
T/C-2	684.8 ± 10.5	610.8 ± 8.5	16.3 ± 1.4	4.0 ± 0.2

## Data Availability

All data that support the findings of this study are available from the corresponding authors upon reasonable request.
